# The Use of Plants That Seal Blood Vessels in Preparations Applied Topically to the Skin: A Review

**DOI:** 10.3390/molecules30091973

**Published:** 2025-04-29

**Authors:** Barbara Hanna Roman, Anna Muzykiewicz-Szymańska, Katarzyna Florkowska, Magdalena Tkacz, Bartłomiej Wilk, Łukasz Kucharski, Agata Madalińska, Anna Nowak

**Affiliations:** 1 Department of Cosmetic and Pharmaceutical Chemistry, Pomeranian Medical University in Szczecin, Powstańców Wielkopolskich Ave. 72, 70-111 Szczecin, Poland; barbara.roman@pum.edu.pl (B.H.R.); anna.muzykiewicz@pum.edu.pl (A.M.-S.); katarzyna.florkowska@pum.edu.pl (K.F.); magdalena.tkacz@pum.edu.pl (M.T.); bartlomiej.wilk@pum.edu.pl (B.W.); lukasz.kucharski@pum.edu.pl (Ł.K.); 2 Students’ Scientific Club at the Department of Cosmetic and Pharmaceutical Chemistry, Pomeranian Medical University in Szczecin, Powstańców Wielkopolskich Ave. 72, 70-111 Szczecin, Poland; madla0103@gmail.com

**Keywords:** vascular skin, blood vessels, medicinal plants, therapeutic compounds, polyphenols, vitamins, carotenoids, saponins

## Abstract

Plants provide valuable compounds that positively influence the health of blood vessels, including those in the skin. Numerous plants exhibit anti-inflammatory, antioxidant, and vasodilating effects, which enhance blood circulation and may promote skin regeneration and suppleness. Botanical species like *Camellia sinensis*, *Chrysanthellum indicum*, *Helichrysum italicum*, *Glycyrrhiza glabra*, *Ginkgo biloba*, or *Artemisia lavandulaefolia* may positively influence the health of cutaneous blood vessels in the skin. The beneficial impact in this context is attributed to various secondary metabolites inherent to these plants, including phenolic acids, flavonoids, vitamins, or saponins, which can subsequently enhance microcirculation, diminish swelling, inhibit telangiectasia, occlude blood vessels, and enhance skin appearance. In addition, the high antioxidant activity of plants is also key here, which helps protect vessels from damage caused by oxidative stress. This article provides an overview of specific plants that may positively influence skin blood vessels, along with a discussion of particular active compounds within these plants that exhibit such effects. These herbs not only improve vascular health but also promote a more youthful appearance. By examining their distinct qualities, we can enhance our comprehension of their synergistic effects on skin vitality and resilience.

## 1. Introduction

Herbal medicines have always been an important part of traditional medicine, and their use in treating various health conditions is also gaining popularity in modern medicine. Knowledge about the health properties of various plants was acquired over the generations and supplemented based on the development of technology (Table 1) [1]. Nowadays, there is a growing interest in medicinal plants and biocompounds obtained from them. Analysis of the way these compounds work helps to ensure the quality and effectiveness of herbal medicines. Developing medical care is starting to include herbal medicines as a support in the treatment of modern diseases. Plants are an extraordinary example of living organisms that are able to produce and store specific secondary metabolites in their tissues (e.g., roots, leaves, fruits, skin, or flowers) [2,3,4]. Bioactive compounds can have direct or indirect therapeutic effects on other organisms. In addition, the concentration of produced biocompounds is influenced by many different factors, such as internal developmental genetic circuits (regulated gene, enzyme) and external environmental factors (light, temperature, water, etc.) [5,6,7,8].

One of the areas in which herbs have a broad therapeutic effect is vascular diseases of the skin, which are a group of diseases affecting blood vessels in the skin and leading to their damage as well as blood flow disorders. They are most often characterized by skin lesions such as spider veins, redness, swelling, or petechiae. Chronic skin inflammation leads to the following diseases: rosacea, telangiectasia, varicose veins (varices), skin purpura (purpura senilis), Raynaud’s disease, Cushing’s syndrome (cushingoid), hemangioma, ulcerative dermatitis, inflammatory vascular disease, or venous ulcer [23,24]. Herbs are a rich source of various active compounds, including flavonoids [25,26], saponins [27], lignans [28], coumarins [29], polyphenols [30], and carotenoids [31,32,33]. Among the plants with properties that strengthen blood vessels, we can mention *Aesculus hippocastanum* [34], *Achillea millefolium* [35], *Ruscus aculeatus* [36], *Ginkgo biloba* [37], *Arnica montana* [38], or *Calendula officinalis* [39]. The biologically active compounds contained in them (e.g., rutin, quercetin, kaempferol, polyphenol acids) have anti-inflammatory, antioxidant properties and stimulate circulation, supporting the condition of the circulatory system [40,41,42,43]. Topical application of preparations containing plant extracts rich in biocompounds to the skin is intended to improve the overall appearance of the skin by strengthening, among others, blood vessels in the skin [44,45,46].

Strengthened and protected skin is a reflection of the health of the body, which is always a subject of human concern. Modern scientific research confirms the effectiveness of many plants in improving the elasticity of blood vessels, reducing swelling, and supporting their resistance to damage [47,48,49,50]. Herbal preparations for strengthening blood vessels are therefore becoming a valuable complement to conventional therapies in the treatment of diseases related to the circulatory system, such as varicose veins or atherosclerosis [48]. In addition, herbal medicines may have a milder side effect profile than synthetic drugs, although further research is needed in this area [51]. Herbal medicines are gaining importance again recently due to the growing interest in a healthy lifestyle as well as the significant increase in civilization diseases in recent decades. The increasingly faster pace of life, excess responsibilities, stress, environmental pollution, and sleep deprivation as well as unhealthy, highly processed food contribute to the increasingly early development of diabetes [37], obesity [38], cancer [39], mental disorders [40,41], digestive system diseases [42,43], and cardiovascular diseases [44]. At the same time, the growing awareness of leading a healthy lifestyle is causing an increase in interest in functional food, including herbs. The popularity of herbal medicine has contributed to the increased usefulness of herbal preparations in order to enhance the effectiveness of conventional pharmacotherapy during the treatment of cardiovascular system diseases [20,45]. This is related to the common belief that plants have a reputation for being natural and safe products. The presence of various active compounds, primarily flavonoids, contributes mainly to the neutralization of free radicals [46] as well as anti-inflammatory effects [47], antiproliferative effects [48], strengthening of vessel walls [49], and a generally positive effect on blood vessels [50].

All of the abovementioned substances, acting synergistically, improve the condition of blood vessels, support their regeneration, reduce inflammation, and strengthen their structures. Regularly including plant substances with a vessel-sealing effect in the diet can also be an effective strategy in the prevention of circulatory system problems. In this review, we wanted to emphasize the health-promoting effect of plants that are a source of valuable compounds such as flavonoids on the strengthening of blood vessels in the skin.

The species analyzed in this article were selected based on a review of the literature from recent years of research. Available databases such as Google Scholar, PubMed, ScienceDirect, and BazTech were used to select the literature. The selection criteria were mainly key words such as: vascular skin, blood vessels, medicinal plants, therapeutic compounds, polyphenols, vitamins, carotenoids, and saponins.

## 2. Natural Substances of Plant Origin with a Sealing Effect on Blood Vessels

**Figure 1:**
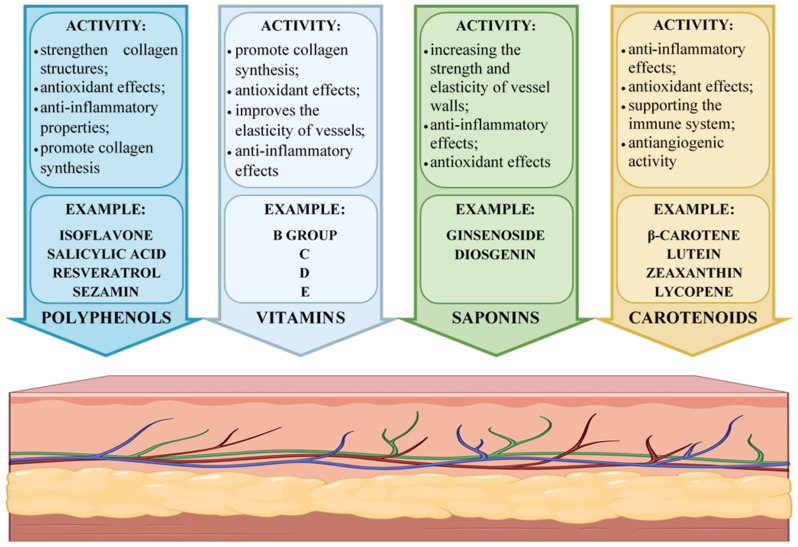
The main groups of compounds affecting the condition of blood vessels in the skin.

Medicinal plants provide a diverse array of bioactive chemicals that are utilized to cure various conditions while resulting in fewer consequences than synthetic pharmaceuticals. Since the beginning of time, humanity has sought to utilize herbs to enhance and preserve health. Enhancing blood vessel strength is crucial for maintaining circulatory system health, and numerous classes of phytochemicals contribute to the flexibility and integrity of vascular walls. Natural compounds, including polyphenols, vitamins, saponins, and carotenoids, are crucial for safeguarding blood vessels from injury and inhibiting the onset of vascular skin illnesses (Figure 1) [52,53,54,55,56].

### 2.1. Polyphenols

**Figure 2:**
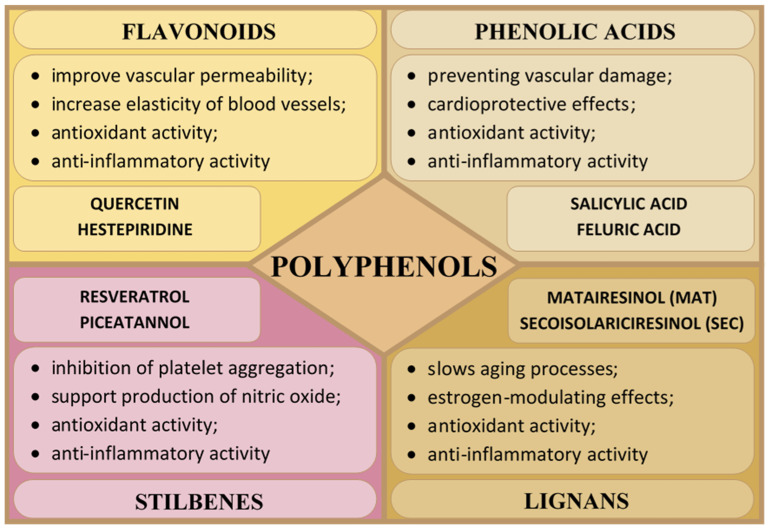
The main groups of polyphenols.

The largest group of compounds with a health-promoting effect on blood vessels are polyphenols. They support the function of the endothelium—the layer of cells lining the inside of blood vessels. They also have a relaxing effect on vessels, lowering blood pressure and reducing the risk of cardiovascular diseases [57]. Polyphenols are secondary metabolites of plants (fruits, vegetables, herbs, and medicinal plants) produced in response to environmental stress or injury [58]. They play a protective role against fungi, bacteria, insects, and UV radiation. They are also responsible for the characteristic astringent and bitter taste of plants. Polyphenols are produced via the shikimate and/or acetate biosynthesis pathways, which contain one or more hydroxyl groups attached to a benzene ring, i.e., phenolic units (C_6_H_6_O) [57,58,59]. Based on the number and structure of the phenolic unit bonds, polyphenols can be divided into four main groups: flavonoids, phenolic acids, stilbenes, and lignans, which are characterized by various physical, chemical, and biological properties (Figure 2) [60,61].

#### 2.1.1. Flavonoids

**Figure 3:**
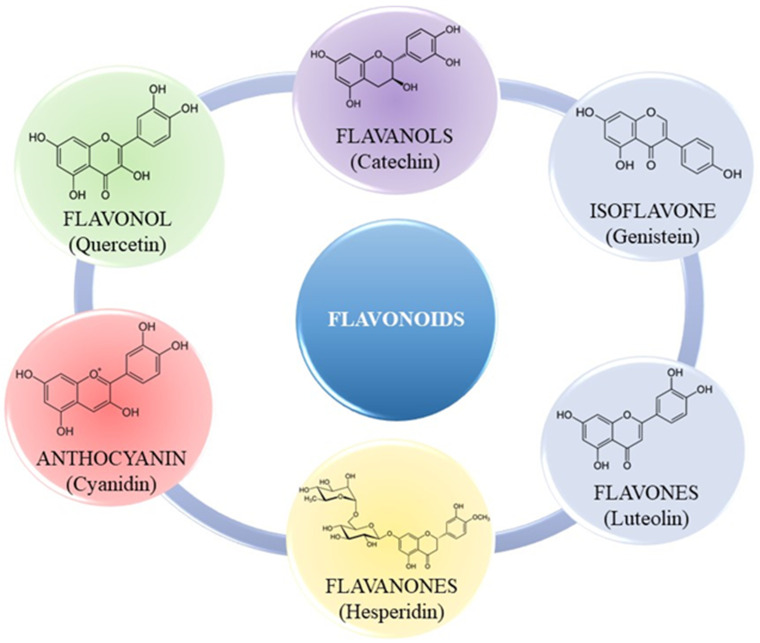
General class of flavonoids.

Flavonoids are regarded as a crucial component in numerous cosmetic, nutraceutical, pharmacological, and medicinal applications. Plants manufacture them as secondary metabolites to provide color and to offer protection against UV radiation, fungi, and insects [58]. They may function as plant hormones, growth regulators, or inhibitors of several enzymatic reactions [62]. Flavonoids consist mainly of a benzopyrone ring containing phenolic or polyphenolic groups at various positions. They are synthesized in plants via the phenylpropanoid pathway [63]. They are primarily distinguished by antioxidant, anti-inflammatory, antimutagenic, and anticancer properties [64,65,66,67,68]. Flavonoids are potent antioxidants that enhance collagen structures in blood vessel walls, improve their permeability and elasticity, and protect against the harmful effects of oxidative stress, which can weaken blood vessels [44,69]. The aforementioned biological effects are contingent upon the specific flavonoid, its potential mechanism of action, and its bioavailability. These characteristics have facilitated research aimed at elucidating the mechanisms of flavonoid activity. Various subgroups of flavonoids have been identified based on the chemical structure, degree of unsaturation, and oxidation of the carbon ring, including: isoflavones, flavonols, anthocyanins, flavanols, flavones, and flavanones (Figure 3) [44,70,71,72].

Flavonoids, primarily obtained from plant foods, are also known as dietary flavonoids. Their bioactivity is contingent upon the mechanisms of absorption and bioavailability. Consequently, enhanced efforts are underway to extract these chemicals from diverse plant sources [8,71,73]. In evaluating the influence of flavonoids on human health, the term bioavailability refers to the degree to which a compound is absorbed and utilized by the body. We can differentiate between “bioavailability” and “bioaccessibility”. “Bioavailability” refers to the fraction of ingested flavonoids that have been absorbed and metabolized by standard physiological processes. “Bioaccessibility” refers to the fraction of flavonoids that is liberated from the food matrix and available for absorption in the small intestine [74,75]. In the human body, ingested flavonoids undergo phase I and phase II transformations. Phase I transformations include oxidation, reduction, and hydrolysis, which lead to the production of flavonoids in the form of aglycones. Phase II transformations, which take place in the liver and intestines, are conjugation reactions, which produce various metabolites, i.e., methyl, glucuronic, and sulfate derivatives [76,77]. Examples of sources of flavonoids are presented in Table 2.

#### 2.1.2. Phenolic Acids

**Figure 4:**
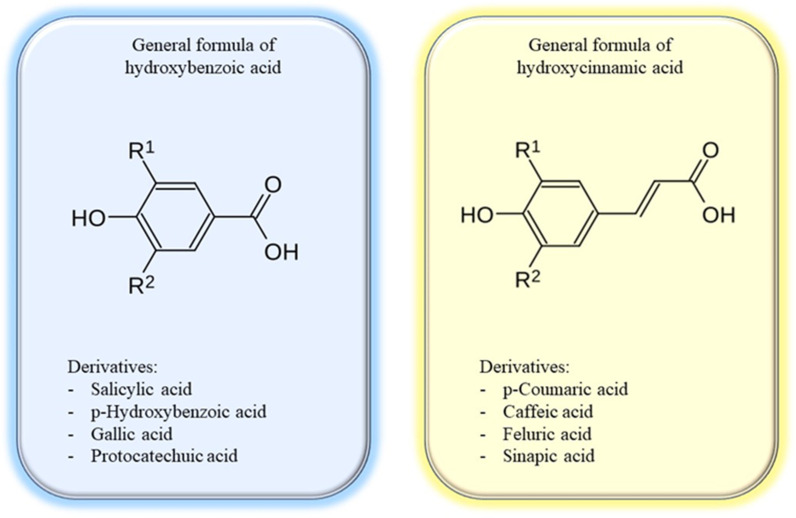
Two general classes of phenolic acids.

Phenolic acids are among the most recognized categories of bioactive chemicals found in many plant sources, including fruits, vegetables, spices, and grains. These are organic compounds that incorporate a phenolic group (–OH) and a carboxyl group (–COOH) inside their structure. The predominant phenolic acids are hydroxybenzoic and hydroxycinnamic acids (Figure 4). Benzoic acids can be biosynthesized via the intermediate shikimic or phenylpropanoid routes. Metabolically, they serve as precursors for primary and secondary metabolites in plants, including phytohormones, defence chemicals, and the manufacture of structural components of the plant cell wall [80]. These compounds are aromatic secondary metabolites of plants that contribute to the distinctive organoleptic properties of food (color, taste, astringency, and pungency) and possess numerous health benefits, including antioxidant, anti-inflammatory, immunoregulatory, antiallergic, antiatherosclerotic, antimicrobial, antithrombotic, cardioprotective, hepatoprotective, neuroprotective, anticancer, and antidiabetic effects [80,81,82,83,84,85,86]. Phenolic acids demonstrate antioxidant activity comparable to that of antioxidant vitamins, including vitamins C and E [84,87]. Rosmarinic acid is the most potent antioxidant among all hydroxycinnamic acid derivatives [88]. Chlorogenic acid is one of the most accessible molecules compared to other phenolic acids due to its abundance in various foods and beverages (coffee) [84]. In Table 3 are presented examples of sources of phenolic acids.

#### 2.1.3. Stilbenes

**Figure 5:**
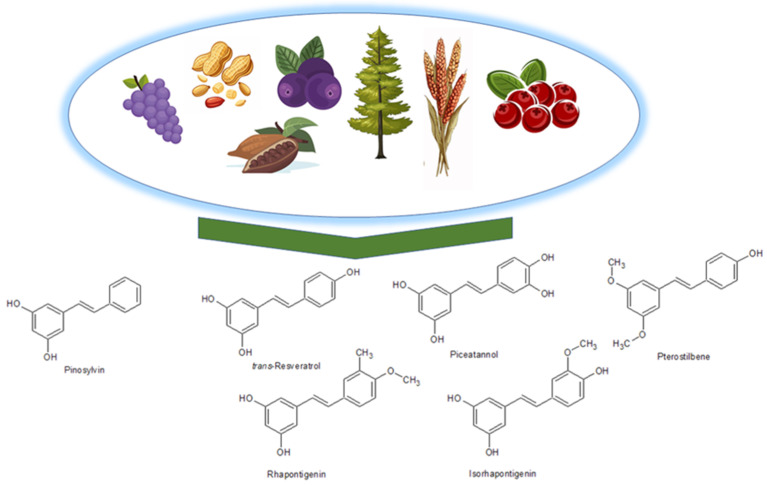
The sources of stilbenes.

Stilbenes are a category of organic compounds characterized by a carbon framework including two benzene rings linked by a carbon–carbon double bond (C=C). They are produced through the phenylpropanoid route, wherein stilbene synthase (STS; EC 2.3.1.95) facilitates the synthesis of simple monomeric stilbenes (e.g., resveratrol, pinosylvin, or piceatannol) from coenzyme A esters of cinnamic acid and three malonyl-CoA units in a singular process. Thereafter, simple stilbenes may undergo glycosylation, methylation, or prenylation through the activity of particular enzymes [90]. These chemicals are extensively found in nature, particularly in plants such as grapes, sugar cane, walnuts, and berries, including blueberries (Figure 5). They execute several biological activities. In plants, they serve as a protective agent against infections due to their antiviral and antifungal characteristics. Moreover, they demonstrate potent antioxidant and anti-inflammatory characteristics, which aid in diminishing inflammation in blood vessels and neutralizing free radicals that may harm endothelial cells and contribute to the onset of cardiovascular illnesses. They facilitate the synthesis of nitric oxide (NO), essential for preserving optimal endothelial function. Nitric oxide influences vasodilation and enhances blood circulation. Stilbenes can inhibit excessive platelet aggregation, hence diminishing the likelihood of thrombus formation and vascular obstructions. Furthermore, they safeguard the skin from ultraviolet B (UVB) radiation, inhibit melanogenesis, promote collagen synthesis, and have anticancer properties, which is why they have been appreciated for their protective effects in the context of heart disease and the aging process [49,90,91,92,93,94,95,96,97,98]. Resveratrol, a prominent representative of stilbenes, is mostly sourced from grapes and red wine [99].

#### 2.1.4. Lignans

**Figure 6:**
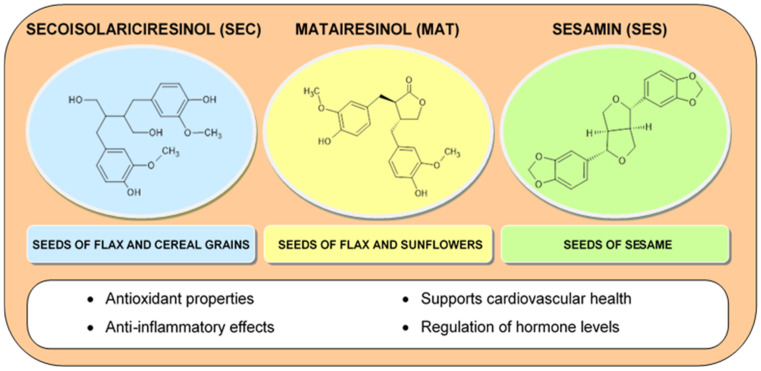
Properties of specific lignans for the skin.

Another noteworthy group of compounds are lignans belonging to diphenolic compounds, which consist of two phenylpropanoid fragments. In addition, their structural skeleton resembles steroids, which is why they are often classified as phytoestrogens. Among them, there are eight structural groups (dibenzocyclooctadiene, dibenzylbutyrolactone, dibenzylbutyrolactol, dibenzylbutane, aryltetralin, arylnaphthalene, furofuran, and furan). Aryltetralin derivatives are often the subject of research due to their interesting antibacterial and antiviral properties. Examples of lignans found in plants are secoisolariciresinol (SEC) and matairesinol (MAT) in flax seeds and sesamin (SES) in sesame seeds (Figure 6) [100,101,102]. Lignans are characterized by strong antioxidant properties, protecting the skin from free radicals, as well as slowing down the aging process. Thanks to their anti-inflammatory properties, they reduce the occurrence of inflammatory diseases of the blood vessels. Lignans are characterized by estrogen-modulating properties, because they have the ability to bind to estrogen receptors, which may be beneficial in preventing hormonal disorders in women [28,72,103,104,105,106,107,108]. The studies conducted indicate a positive effect of lignans isolated from *Premna serratifolia* or flax seeds on the process of inhibiting melanogenesis, which limits the development of melanoma [109,110,111,112].

### 2.2. Vitamins

#### 2.2.1. Vitamin C

Among the wide range of vitamins, vitamin C has the greatest impact on the health of blood vessels. Due to the inability of the human body to synthesize vitamin C, it must be supplied with a diet containing fruits and vegetables such as citrus fruits, berries, tomatoes, potatoes, and green vegetables [113]. Vitamin C is an organic chemical compound γ-lactone of 2,3-dehydro-L-gulonic acid, belonging to the group of unsaturated polyhydroxy alcohols, also known as ascorbic acid. It occurs naturally as a compound with the L configuration in the side chain and the D configuration of the furan system. Citrus fruits, berries, tomatoes, potatoes, and green vegetables are excellent sources of vitamin C. Ascorbic acid is soluble in water and is completely absorbed in the small intestine [113,114]. Vitamin C plays a key role in the synthesis of collagen, which is the basic building block of blood vessels. Vitamin C is needed for the hydroxylation of the proline residue present on procollagen, creating a triple helix of mature collagen. Therefore, vitamin C improves the entire structure of vessels, increasing their resistance to damage and improving blood circulation [45,115]. It supports antioxidant protection against UV radiation-induced photodamage, as well as inhibits the development of melanoma [116,117,118]. Vitamin C is the primary complement to vitamin E and works synergistically with vitamin E to protect against oxidative damage [119]. Vitamin C is successfully used in the treatment of hyperpigmentation [120]. Moreover, in the human body it increases the efficiency of iron absorption from the diet [113,121,122].

#### 2.2.2. Vitamin E

Another vitamin with a significant impact on the condition of blood vessels is vitamin E, which, like vitamin C, acts as a strong antioxidant. It protects blood vessels from damage caused by free radicals [123]. The chemical structure of vitamin E is based on a two-ring skeleton of 6-chromanol and a side chain composed of three isoprene units. Vitamin E is synthesized via the isoprenoid biosynthesis pathway, which is part of the mevalane pathway. Some of the main sources of this vitamin are, in particular, the seeds of oil plants such as soybeans and sunflowers, as well as olives, almonds, pistachios, pumpkin seeds, hazelnuts, spinach, broccoli, kale, avocado, kiwi, mango, and buckwheat [123,124,125,126]. Vitamin E is a strong lipophilic antioxidant that protects membranes from lipid peroxidation and consequently from oxidative damage, which strengthens blood vessels. It has an effective anti-inflammatory potential for inflamed skin after topical application [123,125,127]. Vitamin E supports the proper functioning of the endothelium, improving its ability to relax the vessels and improving elasticity, therefore helping to regulate blood pressure. This vitamin prevents excessive platelet aggregation, which reduces the risk of clots as well as the deposition of cholesterol in the walls of the vessels, narrowing the vessels and impeding blood flow, which is key in preventing atherosclerosis [128,129]. Additionally, studies have confirmed its anticancer, photoprotective, and antiaging properties [130,131,132,133,134,135]. Moreover, the use of vitamin E as a component in bioadhesive films helps improve wound healing and skin regeneration [136].

#### 2.2.3. Vitamin D

Vitamin D is a fat-soluble steroid organic chemical compound. Under the influence of UV radiation, it is converted in the skin from 7-dehydrocholesterol. Its sources are cod liver oil, cheese, egg yolks, mackerel, salmon, tuna fish, and beef liver [137]. The involvement of vitamin D receptors in inflammation additionally facilitates the reduction of atherogenesis by reducing proinflammatory factors and increasing anti-inflammatory factors, thus preserving the endothelial function of vascular muscle cells [137,138,139].

#### 2.2.4. Vitamins from the B Group

**Figure 7:**
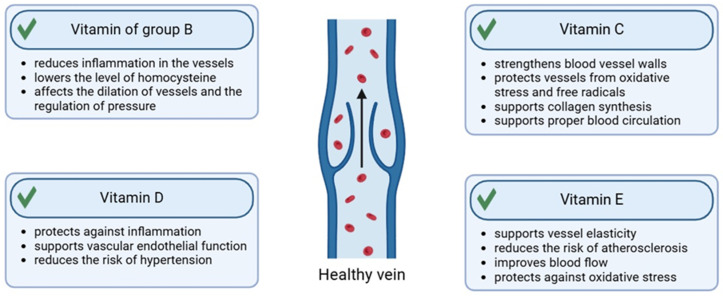
Positive effects of vitamins on blood vessels in the skin.

The use of preparations with a complex of B vitamins has a health-promoting effect on the condition of the skin as well as the blood vessels in it, supporting their function, elasticity, and protection against damage. Vitamins B3 (niacin) and B5 (pantothenic acid) support the improvement of endothelial function, having a positive effect on the dilation of vessels and improving blood flow. In addition, they lower the level of bad cholesterol (LDL) and increase the level of good cholesterol (HDL), which reduces the risk of atherosclerosis [52,140]. Vitamin B2 (riboflavin) helps maintain healthy blood vessel walls by supporting metabolic processes that help regenerate endothelial cells. It also acts as an antioxidant, reducing oxidative stress that can damage vessels. Pyridoxine (vitamin B6) helps reduce homocysteine, an amino acid, elevated levels of which can damage blood vessel walls and increase the risk of cardiovascular disease. Vitamin B9 (folic acid) also reduces homocysteine levels. It also supports the regeneration and health of blood vessel walls. Biotin (vitamin B7) supports the metabolism of carbohydrates, fats, and proteins, helping to maintain metabolic balance and thus the health of blood vessels [140,141,142,143]. Vitamin B12 (cobalamin) supports homocysteine metabolism, similarly to vitamin B6 and folic acid, which leads to a reduction in homocysteine levels and protection of blood vessels from damage. Local application of a preparation with vitamin B12 to the skin has anti-inflammatory, antifibrotic, and antiradiation effects [144,145]. It is worth mentioning that the above vitamins also participate in inhibiting angiogenesis [140,145] (Figure 7). 

### 2.3. Saponins

**Figure 8:**
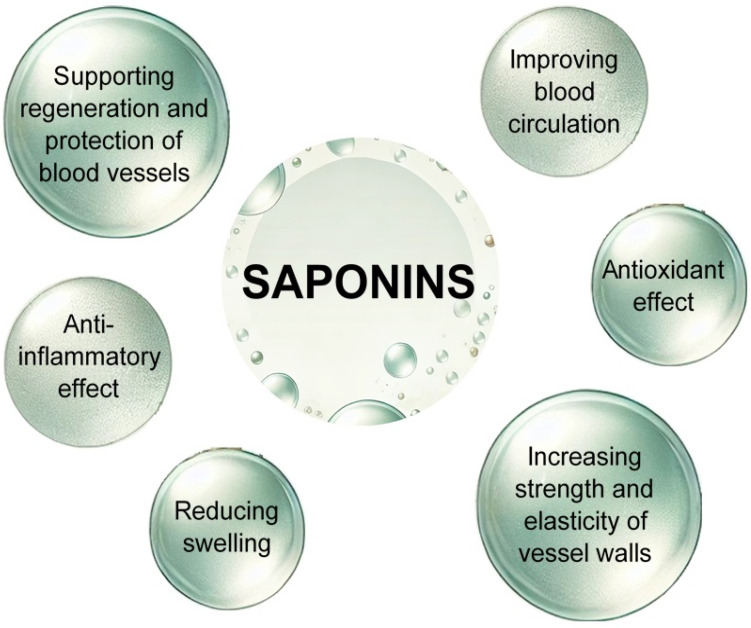
Positive effects of saponins on the blood vessels in the skin.

Saponins are bioactive phytochemicals that enhance the functionality of cutaneous blood vessels. They are categorized as glycosides, wherein the role of the hydrophobic aglycone is fulfilled by sapogenin, characterized by either triterpene or steroid properties. The biosynthesis process of saponins is intricate and comprises multiple stages. Saponins originate from isoprenoid precursors, which are enzymatically converted into squalene and subsequently biosynthesized into sapogenins. In the subsequent phase, sugar moieties, such as glucose and rhamnose, can be conjugated to sapogenin by glucosyltransferase enzymes. Ultimately, triterpene saponins (e.g., ginsenosides) or steroid saponins (e.g., diosgenin) are obtained [27,54,146]. The sources of saponins are *Quillaja saponaria* (soapbark), *Sapindus mukorossi* (soapnut), *Saponaria officinalis* (soaproot), *Saponaria officinalis* L. (soapwort), *Avena sativa* L. (oat), *Aesculus hippocastanum* L. (horse chestnut), *Chenopodium quinoa Willd*. (quinoa), *Vaccaria hispanica* (Mill.) *Rauschert* (cow’s weed), *Glycine max* (L.) *Merr*. (soybean), and *Panax notoginseng* (Chinese ginseng) [147,148]. Saponins are distinguished by their foaming properties akin to soap, which lower surface tension, and from a therapeutic perspective, they exhibit antibacterial, diuretic, expectorant, or emetic actions. Regarding the effects on cutaneous blood vessels, they facilitate the reduction of edema, enhance blood circulation, augment the strength and elasticity of vessel walls, and, through their anti-inflammatory and antioxidant properties, promote the regeneration and safeguarding of blood vessels (Figure 8) [25,27,54,149,150].

### 2.4. Carotenoids

**Figure 9:**
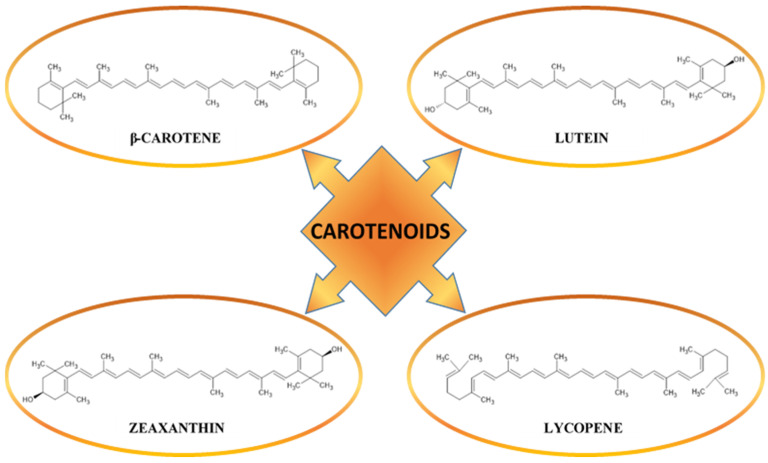
Structures of common carotenoids.

Carotenoids are a group of organic compounds characterized by a chemical skeleton structure based on two cyclohexyl rings connected by a long carbon chain, in which there is a system of a series of conjugated carbon–carbon double bonds. The carbon chain is formed by isoprene units containing five carbon atoms. Due to the characteristics of the structure, carotenoids are classified as mixed cyclic–linear polyenes [151]. Carotenoids can be divided into two groups: carotenes and xanthophylls (Figure 9). A characteristic feature of carotenes is their structure containing only carbon and hydrogen atoms. Unlike carotenes, xanthophylls contain oxygen atoms in their structure, hence they are oxygen derivatives of carotenes [55]. Carotenoids in plants act as pigments responsible for the intense colors of fruits, vegetables, as well as plant parts: flowers and leaves. However, in order for carotenoids to give a color from yellow to red, they must have at least seven double bonds in their alkyl chain, which is why substances such as phytoene or phytofluene, which have only three and five double bonds, are colorless [152]. Other functions performed by carotenoids in plants include: participation in the photosynthesis process, protection against UV radiation and oxidative stress, support of defense processes, influence on the hormonal balance of plants, and as precursors of vitamin A [32,153]. Thanks to their various properties, including antioxidation, carotenoids have a key impact on human health, including by participating in the protection of cells against oxidative stress and UV radiation, anti-inflammation, and supporting the immune system. Supplied in the diet, they contribute to the prevention of many diseases, such as heart disease, cancer, or obesity [151]. Carotenoids such as α-, γ-, β-carotene, lutein, zeaxanthin, lycopene, and their isomers taken with food accumulate in the skin, where they serve living cells, including blood vessels, with protection against oxidation, neutralizing free radicals (FRs) (mainly reactive oxygen species (ROS)) [55,56]. In addition, studies show that carotenoids inhibit the process of vascular formation (antiangiogenic activity) [154,155]. In Table 4 are presented examples of sources of carotenoids.

In Table 5 we have included the effects of individual active compounds found in plants that have a positive effect on the condition of blood vessels in the skin.

## 3. Plants in the Treatment of Vascular Skin

One of the areas in which herbs have a broad therapeutic effect is vascular diseases of the skin, which are a group of diseases affecting blood vessels in the skin and leading to their damage as well as blood flow disorders (Table 6).

They are most often characterized by skin lesions such as spider veins, redness, swelling, or petechiae, while chronic skin inflammations manifest themselves, among others, by: rosacea, telangiectasia, varicose veins (varices), purple skin (purpura senilis), hemangioma, or bruises. For example, one of the main skin problems is the occurrence of spider veins (telangiectasis). These are dilated thin veins located just below the surface of the skin that appear as dark red to bluish veins with many branches. Due to the weaker connective tissue, this defect more often affects women’s skin than men’s. These changes are most often located on the skin of the legs and face, especially around the nose, and are generally a cosmetic defect, but in some cases they may indicate general venous insufficiency. Telangiectasias are also one of the effects of photoaging of the skin. The breakdown of collagen and elastin induced by sun damage (solar elastosis) makes veins more visible as they approach the skin’s surface. Other factors predisposing to the formation of telangiectasias include excessive alcohol consumption, smoking, a sedentary lifestyle, obesity, and pregnancy [41]. Vascular skin changes can also occur in the course of various skin diseases, as well as occur as a manifestation of systemic diseases and as a cosmetic defect. One of the most well-known vascular skin diseases is rosacea. It is a chronic, inflammatory skin disease of multifactorial etiology. Skin symptoms of rosacea include, among others, redness, facial erythema, inflammatory papules and pustules, and telangiectasias. Despite the etiology not being fully explained, the participation of factors such as congenital dysregulation of the immune system and vascular–neuronal dysregulation and damage induced by UV radiation is emphasized [33].

Damaged blood vessels can also cause bruising, which is defined as a red, blue, or black discoloration of the skin caused by blood leaking from damaged blood vessels. Bruising is a very common side effect of cosmetic procedures [170]. Bruising is caused by many surgeries and procedures outside of dermatology and cosmetics, such as plastic surgery [171]. Vascular skin changes can also occur in the course of viral infections, such as COVID-19. They can take on a variety of appearances, such as erythematous rash, violaceous macules with a “porcelain-like” appearance, livedo, non-necrotic and necrotic purpura, chilblains which may also be accompanied by Raynaud’s phenomenon, and eruptive cherry angioma. The probable mechanism of skin vascular changes is related to angiotensin-converting enzyme 2 (ACE2), which is the cellular receptor for the COVID-19 virus. This method of the COVID-19 virus’s entrance into human cells causes the accumulation of angiotensin II. Excess angiotensin II may lead to acute lung injury and vascular dysfunction, including vasoconstriction, increased vascular permeability, and aberrant cardiac remodeling [172].

### 3.1. Camellia Sinensis

*Camellia sinensis* is a plant from the tea family (*Theaceae*). This plant is an evergreen shrub, cultivated mainly in China, India, and Japan [173]. The leaves are lanceolate, shiny, and dark green. The raw material used in medicine is young leaves, which are subjected to minimal fermentation after harvesting. They contain polyphenols, caffeine, theanine, and vitamins A, C, and E [174,175]. *C. sinensis* is known for its antioxidant, anti-inflammatory, and cardiovascular supporting properties [176]. The main secondary metabolites contained in green tea responsible for sealing blood vessels are considered to be numerous small catechins, which include (−) epigallocatechin gallate (EGCG), (−) epicatechin gallate (ECG), (−) epicatechin (EC), and (−) epigallocatechin (EGC), in addition to epiisomers such as (+) gallocatechin gallate (GCG), (+) catechin gallate (CG), (+) gallate (GC), and (+) catechin, among which EGCG occurs in the largest amount [42]. Studies have proven the efficacy of this plant in the treatment of patients prone to skin diseases manifested by erythema and telangiectasias, and EGCG, belonging to the polyphenol group, is mentioned as the main substance responsible for this action [157]. Domingo et al. [157] conducted a small randomized, double-blind, split-face trial using a cream containing 2.5% *w*/*w* EGCG. Four volunteers (three men and one woman) aged 40–59 years with significant erythema and telangiectasias on the face applied a cream containing EGCG to one side of the face and a control cream to the other side for 6 weeks. The results of a skin biopsy after the study had been completed showed the inhibitory effect of EGCG on angiogenic growth and transcription factors, vascular endothelial growth factor (VEGF,) and hypoxia-inducible factor-1 (HIF-1). Although the assessment of skin redness performed with a chromometer did not show a clinically observable difference, the results of immunohistochemical studies confirm the antiangiogenic potential of EGCG. Therefore, it is suggested that local application of EGCG may serve as a potential agent in the prevention of telangiectasias [157]. Another study evaluated the effect of topical and oral green tea supplementation on clinical and histological features of photoaging. Forty women with moderate photoaging received a 10% *C. sinensis* cream and 300 mg of oral green tea supplementation twice daily as well as placebo for 8 weeks. Patients applied tea extract cream twice a day to the face and arms. After the study was completed, treated patients showed histological improvement in elastic tissue content. Telangiectasias also improved in the *C. sinensis* group, but without significant differences compared with the control. The authors suggest that the 2-month study period may not be sufficient time to achieve clinically visible skin changes in this regard [158]. Among the mechanisms of sealing blood vessels through polyphenols contained in *C. sinensis*, the protective effect of these compounds on hyaluronic acid and inhibition of histamine release are mentioned. In addition, their effect on adrenaline activity and prevention of oxidation of this hormone is also emphasized. Adrenaline constricts blood vessels, and its low level leads to their weakening, through shorter but more frequent contractions. In addition, the polyphenols of this plant may also affect the mechanisms leading to improved blood flow, such as inhibition of the proteolytic activity of thrombin and inhibition of the tyrosine kinase activity of Syk and Lyn [177]. Another factor is the strong anti-inflammatory effect of polyphenols, which consequently significantly improves skin microcirculation. This effect is associated with the very high antioxidant activity of these compounds, which, by protecting cells, slows down their inflammatory processes while disturbing skin microcirculation. Additionally, EGCG significantly inhibits the production of cyclooxygenase (COX)-1 and thromboxane synthase (TXAS) in platelets, which are the two main enzymes responsible for platelet aggregation [178].

### 3.2. Chrysanthellum Indicum

*Chrysanthellum indicum* belongs to the Asteraceae family and is a perennial plant found in Asia, especially in China and India. It grows in wet meadows pastures and along roads. It reaches a height of 20–50 cm [178]. The medicinal raw material is the whole herb and flowers, rich in flavonoids, saponins, phenolic acids, and essential oils, while the main components of the essential oil are camphor, borneol, camphene, α-pinene, p-cymene, and 1,8 cineole [3]. The plant has anti-inflammatory, hepatoprotective, and antiviral properties, thanks to which it has been used in traditional and modern medicine [3,4,179]. Rigopoulos et al. [159] conducted a 12-week multicenter randomized, double-blind, parallel group, placebo-controlled study comparing a cream containing 1% extract of *Ch. indicum* vs. placebo applied twice a day. The study included 246 people aged 18–80 years. The study group consisted of 91 women and 34 men, while the control group consisted of 85 women and 36 men. The researchers observed a 41.3% reduction in erythema score (the placebo rate was 32.5%). The authors postulate that *C. indicum* contained in the cream under study exhibits vitamin P properties that may be interesting in reducing or preventing vascular disorders and, in particular, microcirculation disorders in patients with rosacea [159].

### 3.3. Helichrysum Italicum

*Helichrysum italicum* belongs to the aster family (*Asteraceae*). It occurs in the Mediterranean basin in dry, sandy, and rocky areas. The plant shows high adaptation to environments characterized by water deficiency, because it naturally occurs in alkaline, dry, sandy, and poor soils, at altitudes from sea level to 2200 m above sea level [180]. It is a subshrub with stems that are woody at the base reaching 30–60 cm in height, with narrow, silver-gray leaves and small yellow, fade-resistant flowers gathered in corymbs [180]. The raw material is the flowers and herb, rich in essential oils, containing, among others, neryl acetone and α-pinene as well as flavonoids [181]. *H. italicum* has anti-inflammatory, antioxidant, and skin-regenerating properties, which are used primarily in cosmetology [180]. Hettwer et al. [41] described the results of a double-blind, placebo-controlled in vivo study conducted on a group of 43 women aged 30–65 years (mean 55.0) after using cosmetic preparations containing an aqueous extract of *H. italicum* for 28 days. The authors state that the mechanism of action of this preparation is based on the inhibition of nitric oxide synthase (NOS), which produces nitric oxide (NO). NO affects venous dilation and varicose veins, as well as increasing fluid accumulation in tissues, which results in edema. It is suggested that the use of NOS inhibitors may affect the alleviation of acute and chronic inflammatory reactions of vasodilators in the skin, which will contribute to the reduction of the visibility of telangiectasias and reduction of skin redness and edema. NOS inhibitors may be phenolic acids, such as chlorogenic acid and di-O-caffeoylquinic acids, which were identified in the *H. italicum* extract. In the cited study by Hettwer et al., women applied an emulsion containing 5% active ingredient to the skin of their legs twice a day for 28 days, a gel containing 3% active ingredient to the eye area, and an emulsion containing 1% active ingredient to the skin of their face. After 14 and 28 days, a significant reduction in the so-called “eye bag” was observed, as well as a decrease in the basic microcirculation of the skin in the swollen area. After 4 weeks, a significant reduction in red spots was observed. An increase in skin resistance to stress induced by thermal stimulation was also observed. Partial inhibition of NOS activity was also confirmed, which resulted in a decrease in the release of NO as well as a significant reduction in the number of telangiectasias on the skin of the legs. The presented studies confirmed the validity and effectiveness of using *H. italicum* extracts in the care of vascular skin [41].

### 3.4. Glycyrrhiza Glabra

*Glycyrrhiza glabra,* known as smooth licorice, belongs to the legume family (*Fabaceae)*. It occurs in southern Europe, western Asia, and the Middle East [182]. It prefers sandy-clay soils in a warm climate. It is a perennial reaching a height of up to 1.5 m with a very well-developed root system [183]. It has feathery leaves, small purple flowers gathered in clusters, and fruit in the form of flattened pods. The medicinal raw material is the root rich in triterpene saponins (glycyrrhizin), flavonoids, coumarins, and polysaccharides [184]. Licorice has anti-inflammatory, antibacterial, antiviral, and protective effects on the mucous membranes of the digestive tract [182,185]. This plant is a valuable source of phenolic compounds, of which glabridin and glycyrrhizin are the best studied. These substances have, among others, antioxidant, anti-inflammatory, and anti-irritant potential [186]. Weber et al. [160] studied a group of over 32 women with mild to moderate rosacea and 20 women with facial redness that was not related to rosacea. The study participants were aged 20–65 years (average 48 years), and the study period was 8 weeks. During the study, four test preparations containing *G. glabra* root extract were applied to the skin at least once daily. The preparations consisted of a cleanser, an SPF 15 facial moisturizing lotion with UVA/UVB coverage containing green concealing pigments, a spot concealer (with pigments), and a night moisturizing cream. After 4 and 8 weeks of daily product usage, the researchers observed significant improvements in the combined panel’s average erythema scores. Improvements in erythema were smaller in the rosacea vs. the non-rosacea group. In addition, the authors confirmed the safety of using preparations containing *G. glabra* root extract in combination with metronidazole drug therapy. The results of this team’s research confirmed the improvement of the general appearance of the facial skin, which improved the quality of life in patients with rosacea and skin redness. Additionally, the authors suggest the validity of using preparations containing this plant as a supportive therapy to metronidazole drug therapy in patients with rosacea [160].

### 3.5. Artemisia Annua

*Artemisia annua* belongs to the aster family (Asteraceae). It comes from Asia, especially from China, where it grows in humid and sunny areas. It is an annual herbaceous plant reaching up to 2 m in height. It has pinnately incised leaves and small yellow flowers gathered in baskets [187,188]. This plant is known for its antimalarial, antiviral, and anti-inflammatory effects. The medicinal raw material is the herb and leaves containing, among others, artemisinin, flavonoids, and essential oils [43,188]. One of the main active substances of this plant used in medicine is artmisinin, used for many years as an antimalarial drug. Several recent studies have shown that this substance can alleviate inflammation and erythema caused by rosacea, which was shown in earlier studies involving animals. In this study, twenty-five male mice were subcutaneously injected with 40 μL of the antibacterial peptide LL-37 in the back once every 12 h for four sessions to create mouse models of rosacea-like inflammation. The animals were randomly and evenly divided into five groups, where the model group was administered saline by gavage, the treatment groups were administered 25, 50, and 100 mg/kg artemisinin solution by gavage, and the positive control group was administered 30 mg/kg doxycycline hydrochloride solution by gavage. Inflammatory responses were observed in the model group as well as increased erythema (3.20 ± 0.84), inflammatory cell count (517.27 ± 99.43), and MPO activity (0.57 ± 0.08) compared with the control group (all *p* < 0.01). The positive control group showed less erythema (1.60 ± 0.89), a lower inflammatory cell count (270.93 ± 124.63), and less MPO activity (0.40 ± 0.05) compared with the model group (*p* < 0.05, 0.01, 0.01, respectively). Moreover, the erythema score, inflammatory cell number, and MPO activity were significantly lower in the 50 (1.80 ± 0.84, 286.00 ± 33.72, 0.43 ± 0.05, respectively) and 100 mg/kg artesunate groups (1.40 ± 0.55, 258.00 ± 36.44, 0.40 ± 0.06, respectively) than in the model group (*p* < 0.05 or 0.01). The study analyzed excised skin tissue, and the tissue structure was observed and the number of inflammatory cells was counted. As a result, the artemisinin group showed significantly reduced erythema score, as well as number of inflammatory cells and myeloperoxidase (MPO) activity, compared to the model group. In addition, the erythema score, the number of inflammatory cells, and MPO activity were significantly lower in the groups of animals administered 50 and 100 mg/kg artemisinin compared to the model group [161]. Wang et al. [162] conducted a study on the efficacy and tolerance of artemether emulsion for the treatment of papulopustular rosacea, where the active substance used was a lipid-based derivative of artmisinin. The study was conducted for 4 weeks with randomization and blinding of the investigator, conducted in parallel groups, with open observation for an additional 8 weeks. The study group consisted of 130 patients with mild to moderate papulopustular rosacea. Patients were randomly assigned in a 1:1 ratio to the artemether emulsion group (1%) or metronidazole emulsion group (3%). The study confirmed the efficacy in reducing erythema, papules, and pustules and tolerability of topical application of 1% artemether emulsion in the treatment of rosacea. At the end of the study, patients in the artemether emulsion group had a comparable rate of successful response and improvement in erythema, but better improvement in papules and pustules between baseline and week 4, compared with patients in the metronidazole emulsion group. Numerically more patients achieved an effective response at week 4 with artemether emulsion (87.1%) than metronidazole emulsion (80.0%) (*p* > 0.05). Patients treated with artemether emulsion had comparable baseline erythema scores (2.45 ± 0.67 vs. 2.42 ± 0.70, *p* = 0.809) and papules and pustules scores (2.11 ± 0.96 vs. 2.32 ± 0.83, *p* = 0.264) but significantly lower papules and pustules scores (0.21 ± 0.52 vs. 0.42 ± 0.83, *p* = 0.001) and comparable erythema scores (0.53 ± 0.88 vs. 0.62 ± 0.88, *p* = 0.999) compared with patients treated with metronidazole emulsion at week 4. The obtained results confirm the efficacy of 1% artemether emulsion in the treatment of papulopustular rosacea in comparison with metronidazole emulsion after only 4 weeks, but its beneficial effect was maintained for an 8-week observation period in contrast to the effects of metronidazole emulsion [162]. Huang et al. tested the activity of *A. annua* oil extract in local in vivo mouse models for both atopic dermatitis and psoriasis. The study showed that *A. annua* oil extract significantly reduced the release of proinflammatory cytokines IL-4 and IL-17A induced by T cell receptors. The study also tested the effect of AN oil extract in local in vivo models of both atopic dermatitis and psoriasis. In the calcipotriol or MC903-AD induced model, *A. annua* oil extract showed a reduction in mouse ear thickness (edema) and several serum cytokines, IL-1β, IL-6, and IgE. In addition, the analyzed b oil extract also effectively alleviated the lesions, significantly reduced the psoriasis area and severity index to 5.4, and inhibited serum inflammatory mediators (IL-6, TNF-α, IL-1β) in the mouse model of imiquimod-induced psoriasis [189].

### 3.6. Aesculus Hippocastanum

*Aesculus hippocastanum* belongs to the horse chestnut family (*Hippocastanaceae*). Adult trees are up to 30 m tall, and their trunk can be about 1 m thick. It has a well-developed root system and a spreading crown. It usually occurs in forests and dark ravines [14]. It grows in the Balkan Peninsula, but as an ornamental plant it can be found in all countries of the temperate zone. Due to its medicinal properties, it is often a component of ointments for varicose veins, hemorrhoids, and hematomas. Horse chestnut infusions are used for baths in the case of problems related to the circulatory system and improper blood clotting [190]. It contains a number of active ingredients, such as quercetin, kaempferol, rutin, and essential oils. The most well-known component of horse chestnut is escin, which is part of a mixture of triterpene saponins [191]. This compound is found in the seeds and bark of horse chestnut, and extracts obtained from them show effective action in venous insufficiency [14,29]. Escin has anti-inflammatory and vasoconstrictive effects [34]. Horse chestnut extracts and escin are used primarily in chronic venous insufficiency. Horse chestnut fruit is a traditional plant raw material with capillary permeability and anti-inflammatory effects [48,192]. The effectiveness of using horse chestnut seed extracts has been confirmed by many studies. First of all, they have a positive effect on diseases of the circulatory system, which are directly related to oxidative stress. In addition, these extracts are used internally and externally to treat chronic venous diseases, such as edema, varicose veins of the lower limbs, heaviness in the legs, as well as frostbite, burns, and inflammation. Escin inhibits prostaglandin synthesis, which has a strengthening, anti-inflammatory, and sealing effect on the walls of blood vessels. They also inhibit the action of lycosomal enzymes, the activity of which negatively affects the condition of veins, causing damage to the capillaries of the mucopolysaccharide layer. In addition, seed extracts can reduce the permeability of electrolytes, water, and proteins through the interstitial tissue. Escin has a positive therapeutic effect on microcirculation as well as the surrounding connective tissues. Such tissues consist of cellular and fibrous components, providing support and cushioning to blood vessels and fibrous tissues. One of the components is hyaluronic acid, which gives the extracellular matrix viscosity. Hyaluronidase activity reduces the viscosity of these tissues, thus reducing their ability to support and cushion. Escin can block the activity of elastase and hyaluronidase in the process of connective tissue degradation as well as reduce the release of inflammatory mediators, including cytokines, and metabolites of arachidonic acid, such as prostaglandins and leukotrienes. An additional effect of this activity is reduced resistance to fluid leakage from capillaries, as a result of violation of the integrity of the extracellular matrix, which consequently results in increased fluid exchange through capillary membranes. However, in addition to the direct effect of the extract from this plant on blood vessels, an additional positive effect is the protective effect on connective tissues that surround capillaries [48,192]. Özkan et al. report that escin contained in horse chestnut penetrates the skin well and may be an alternative for skin lesions caused by chronic venous insufficiency as well as inflammation of soft tissues [193]. In addition, escin and horse chestnut extracts may also accelerate wound healing, among other effects, by sealing blood vessels [194]. They also show an inhibition effect on proinflammatory cytokines IL-6 and IL-8 and reduce vascular permeability leading to edema, which was demonstrated in the studies of Dumitriu et al., who determined the anti-inflammatory effect at the vascular level of some compounds isolated from *A. hypocastanum* through flow cytometry studies of the expression of adhesion molecules (VCAM-1 and ICAM-1) as well as the proinflammatory cytokines IL-6 and IL-8 in the extracellular environment and VEGF. In the study, inflammation was induced by two different stimuli: LPS, a lipopolysaccharide that mimics bacterial infection, and TNF-α, a non-specific stimulus for the acute phase of systemic inflammation. The isolated compounds inhibited the secretion of IL-6 and IL-8 and blocked the proangiogenic factor VEGF but had no significant effect on the expression of adhesion molecules in bacterial inflammation [163].

### 3.7. Potentilla Erecta

*Potentilla erecta* belongs to the *Rosaceae* family. It grows in Europe and Asia in wet meadows, peat bogs, and forests. It is a small perennial reaching a height of 10–40 cm. It has pinnately compound leaves and yellow flowers with four petals [195]. The medicinal raw material is the rhizome, rich in tannins, triterpenes, flavonoids, and phenolic acids [196]. Cinquefoil has astringent, anti-inflammatory, and antibacterial properties and is mainly used in diseases of the digestive tract and in skin inflammations [196] and commonly used for diarrhea. Although there are no reports of clinical studies on the use of *P. erecta* in the therapy of vascular skin, this plant, due to its anti-inflammatory effect, is often recommended in the care of skin with rosacea [15,186,197]. Wölfle et al. [15] confirmed the ability of *P. erecta* extract to constrict blood vessels. The anti-inflammatory and vasoconstrictive properties were similar to those of glucocorticoids. The vasoconstrictive effect of this extract is at least partly mediated by scavenging NO and inhibition of eNOS. Due to the fact that the tested extract did not cause nuclear translocation of the glucocorticoid receptor in HaCaT cells, side effects are not expected. Such an effect is attributed to the main active compound of *P. erecta*, namely ellagitannin agrimoniin [26]. This compound has numerous hydroxyl groups that interact with reactive molecules on proteins or polysaccharides, which consequently leads to tightening of damaged membranes. In addition, this compound has strong antioxidant properties, because it consists of complex structures of aromatic rings as well as conjugated double bonds. Such a structure allows for electron transfer and consequently scavenging of free radicals. Due to its vasoconstrictor effect, it is also suggested that the extract from *P. erecta* may be useful in the local treatment of inflammatory skin diseases, including rosacea [15]. Studies have shown a significant reduction in erythema caused by UV radiation. In a randomized, prospective, placebo-controlled, double-blind study involving 40 healthy volunteers, the effect of 2% *P. erecta* cream (dry matter) was analyzed using an erythema test in comparison with 1% hydrocortisone acetate. Both PO cream and hydrocortisone acetate showed a significant reduction in UV erythema compared to the vehicle. There was no significant difference between PO cream and hydrocortisone acetate cream (*p* = 0.777). Clinical assessment of the test areas revealed no intolerance reactions. In the UV erythema test, the application of the preparation containing *P. erecta* extract significantly reduced the erythema index. The anti-inflammatory effect of the applied cream was comparable to that of the cream with 1% hydrocortisone acetate. A clinical study in atopic patients showed a significant reduction in the partial SCORAD as well as erythema in the test areas. The authors also emphasize that the preparation used was well tolerated by patients and did not show any adverse effects [198,199].

### 3.8. Achillea Millefolium

*Achillea millefolium* belongs to the *Asteraceae* family, which includes up to 140 species. It is widespread in North America, Asia, and northern and central Europe. It occurs less frequently in southern Europe. It usually grows in meadows, fields, and along roads [200,201]. The plant reaches a height of 30–60 cm. It has feathery leaves and small pink or white flowers forming clusters. They are characterized by an intense scent and a tart taste. Common yarrow has long been known for its medicinal properties, although it also serves an ornamental function [200,201]. In medicine, both the flower and the whole herb obtained during the flowering period, i.e., in the months of June–October, are desirable [200]. Common yarrow contains a number of important substances, such as flavonoids (including apigenin derivatives), monoterpenes, fatty and organic acids, amino acids, phenols, saponins, as well as essential oil containing, among others, chamazulene, sabinene, β-pinene, 1,8-cineole, linalool, α-thujone, β-thujone, ocimene, camphor, ascaridole, caryophyllene oxide, β-eudesmol, and α-bisabolol [35,202]. Extracts obtained from this plant exhibit antibacterial, anti-inflammatory, and analgesic effects. Its effectiveness in medicine is mainly related to the presence of essential oils and phenolic compounds [201]. In traditional Chinese medicine, *A. millefolium* has been used as an antihemorrhagic and wound-healing agent, which made it an effective remedy for ulcers, skin diseases (wounds), snakebites, and varicose veins [17]. It is used externally in wound healing and treatment of skin inflammation [16]. Yarrow herb extract is also used in the prevention and treatment of cardiovascular diseases. Flavonoids found in yarrow herb protect against damage to the heart muscle thanks to their multifaceted properties, such as anti-inflammatory, antioxidant, and antiplatelet effects [203,204]. Extracts can increase the proliferation of blood vessel muscle cells and have anti-inflammatory effects, thus reducing the risk of thrombotic complications. *A. millefolium* extract (150 mg/kg) can significantly shorten the time of local bleeding, as shown by the histopathological analysis of the liver of rats (120–220 g) whose wounds were treated directly with a water–alcohol extract of this plant. Application of *A. millefolium* to liver incisions, whether first or second, significantly reduced bleeding time (by 36.1% and 31.9%, respectively). Histopathological analysis showed no signs of toxicity or hepatic damage after 4, 6, and 8 weeks in female rats [16].

### 3.9. Quassia Amara

*Quassia amara*, a member of the *Simaroubaceae* family, comes from tropical regions of South and Central America. It prefers moist tropical forests and soils rich in organic matter. It is a small tree growing to 2–8 m in height [205]. The leaves are pinnately compound, with a shiny surface, and the flowers are intensely red, gathered in clusters. The medicinal raw material is the bark, wood, and extracts containing bitter substances such as quassic acid and indole alkaloids, as well as steroids (stigmasterol, β-sitosterol, and campesterol) and triterpenes (quassine and neoquasine). The plant has antibacterial, antiedematous, antimalarial, and antiparasitic properties [198,205,206]. Ferrari and Diehl [164] assessed the efficacy of topical application of a gel with 4% *Q. amara* extract for 6 weeks in patients with rosacea. The study was conducted on a group of 30 people aged 21–82 with varying degrees of severity of rosacea (I–IV). Patients applied the preparation twice a day for 45 days to the affected areas. Before the study (day 0), during the study (day 15 and 30), and after its completion (day 45), the following parameters were assessed: flushing, erythema, telangiectasias, papules, and pustules. Patients experienced a continuous decline of all assessed parameters within 6 weeks of treatment. The final reductions were 74%, 56%, 50%, 84%, and 100%, respectively, for flushing, erythema, telangiectasias, papules, and pustules. Within 6 weeks of treatment, 37% of patients experienced significant improvement or complete remission, 22% experienced marked improvement, almost 30% moderate improvement, and only 11% of patients did not experience any improvement. In addition, the tolerance of the preparation was excellent in all patients who completed the study. No adverse effects such as itching, swelling, burning, and stinging were reported. Based on the obtained results, the authors postulate that topical Quassia extract can be an effective and safe element of therapy for people with rosacea [164].

### 3.10. Ginkgo Biloba

*Ginkgo biloba* is one of the oldest trees found in the world [47]. Due to its medicinal properties, it has been appreciated for over 2000 years [207,208]. It is native to China, although it is now cultivated in many regions of Europe, America, New Zealand, and India [207,208,209]. It is a deciduous tree belonging to the Ginkgoaceae family [208]. Adult trees have a wide crown and grow to about 30 m in height. They have long, gray shoots that are alternately covered with leaves and show unlimited growth and black-gray dwarf shoots. The leaves are dark green or pale yellow and have a smooth surface and a fan shape. They can also grow on the tips of short shoots. Ginkgo leaf extracts contain flavonoid glycosides, in particular acetylated p-coumaric acid esters (quercetin, kaempferol, and isorhamnetin) and diterpenes (ginkgolides A, B, C, J, and M containing a tert-butyl group) or sequiterpenoids (bilobalide) [209]. *G. biloba* has primarily strong antioxidant properties, which are mainly due to the presence of flavonoids in the leaves. Additionally, these compounds prevent the permeability of blood vessels. This action protects against the formation of hematomas and edemas. In addition, the plant has anti-inflammatory, antiviral, antibacterial, anticancer, antifungal, and wound-healing effects [37,210]. Valuable substances found in this plant, such as terpenoids (ginkgolides, bilobalides), flavonoids, and flavonol glycosides, have anti-inflammatory effects, which have been linked to antiradical and antilipoperoxidative effects in experimental fibroblast models. Ginkgo leaves are also believed to change skin microcirculation, reducing blood flow at the capillary level and inducing vasomotor changes in the arterioles of the subpapillary plexus of the skin. Together, these changes can lead to a reduction in skin redness [211]. Ginkgolide B contained in *G. biloba* is an antagonist of platelet-activating factor (PAF) and an intracellular mediator involved in the platelet aggregation process and also has antioxidant effects [209]. Some secondary metabolites contained in *G. biloba* have an influence on the formation of new blood vessels. Hu et al. suggest that kaempferol is a key component here. In their studies, kaempferol was shown to bind to vascular endothelial growth factor (VEGF), probably in the heparin-binding domain of VEGF, which enhanced the angiogenic functions of VEGF in different culture models. This compound enhanced VEGF-induced cell motility in human umbilical vein endothelial cells (HUVECs), as well as subintestinal vessel sprouting in zebrafish embryos and microvessel formation in the rat aortic ring. Kaempferol application strongly potentiated VEGF-induced phosphorylations of VEGFR2, endothelial nitric oxide synthase (eNOS), and extracellular signal-regulated kinase (Erk) in a time- and concentration-dependent manner and, in parallel, the expression of matrix metalloproteinases (MMPs) MMP-2 and MMP-9. In addition, the potentiation effect of kaempferol was revealed in VEGF-induced migration of skin cells and monocytes. Taken together, the results suggested the pharmacological roles of kaempferol in potentiating VEGF-mediated functions should be considered [165].

### 3.11. Artemisia Lavandulaefolia

*Artemisia lavandulaefolia,* a member of the *Asteraceae* family, is a species native to East Asia, particularly China and Korea [212]. It grows in dry areas and forests but can also survive in seasonally flooded areas. It is a perennial herb growing up to 1 m tall. It has aromatic, pinnately divided leaves and small yellow flowers [213]. The raw material is the leaves and herb containing essential oils (thujone, camphene) and phenolic compounds. The plant has antiparasitic, antibacterial, anti-inflammatory, and analgesic effects [2,212,214,215]. Roh et al. [166] examined the effect of chlorogenic acid isomers isolated from *A. lavandulaefolia* on the activity of kallikrein protease 5 (KLK5). Increased KLK5 activity in rosacea generates peptide LL-37 by cleaving cathelicidin, which triggers cutaneous inflammation and erythema. The KLK5 inhibitors are considered therapeutic agents that improve the basic pathophysiology and clinical symptoms of rosacea. In patients with rosacea, abnormal activation of cathelicidin by LL-37 due to excessive KLK5 leads, among other effects, to erythema. In a study, isochlorogenic acids A and C were found to regulate the activation of cathelicidin by LL-37 by inhibiting the activity of KLK5, which contributes to the regulation of immune cell and blood vessel functions in rosacea. As a result, these isomers influenced immunomodulation and may improve erythema in rosacea lesions. However, further clinical studies are needed to confirm the therapeutic efficacy of isochlorogenic acids A and C [166]. In other studies, iso-chlorogenic acid significantly reversed ear swelling in mice caused by dermatitis and chronic itching [216]. In other studies, this plant has been shown to inhibit angiogenesis in vitro, which was confirmed by the effect of *A. lavandulaefolia* extract on the formation of blood vessel networks of human umbilical vein endothelial cells (HUVECs). The authors showed that treatment with the extract of this plant inhibited the formation of HUVEC tubes without any effect on HUVEC viability. Furthermore, the extract inhibited HUVEC migration and invasion. In the absence of the analyzed extract, HUVECs formed capillary-like networks, whereas the tube-like structure was suppressed in the presence of *A. lavandulaefolia* extract in HUVECs [2].

### 3.12. Calendula Officinalis

*Calendula officinalis* is a plant belonging to the *Asteraceae* family. It naturally grows in the Mediterranean and central European regions [217]. It does not have strict soil requirements, which makes it easy to maintain [218]. It can therefore be found in various parts of the world, as it is cultivated as an ornamental plant and for cosmetic and medicinal purposes [194]. It is practically an unbranched, fragrant perennial that reaches a height of about 80 cm. It has elongated leaves. The high content of carotenoids, which are natural pigments, provides the orange color of its flowers [218,219]. They are usually about 4–5 cm in diameter and are surrounded by bracts arranged in two rows. In favorable conditions, the flowering period will last the whole year. The fruit is a so-called achene with a curved, arched shape [218,220]. Due to its medicinal properties, it has been valued since the 12th century as a herbal remedy used for burns or wounds. Extracts from the flowers and leaves of marigold have antioxidant and antibacterial properties and also have a regenerative effect on blood vessels [217,221]. They are used in the production of ointments, balms, creams, and gels [221]. Marigold is a plant rich in many active ingredients, which, in addition to carotenoids, also include, among others: saponins, amino acids, tocopherols, essential oils, and phenolic acids (e.g., caffeic, ferulic, chlorogenic) [163,197]. This plant is used topically as a natural anti-inflammatory medicine as well as for faster healing of bruises, first-degree burns, boils, and rashes [222]. It is also a very popular raw material used for poorly healing wounds. For example, Shafeie et al. analyzed the effect of different concentrations of a gel containing *C. officinalis* extract on histological and biomechanical changes in rat skin. The animals were randomly divided into three groups, namely the control group, placebo group, and group treated with the analyzed extract. The treatment group received daily local application of 5%, 7%, and 10% gel containing ethanolic extract of *C. officinalis,* the placebo group received daily local application of the base gel, and the control group received no treatment. The wounds of animals treated with the preparation containing the analyzed plant had no exudate during this time and for the next 14 and 21 days. Moreover, very few inflammatory cells were observed after 21 days of the study. The number of fibroblasts decreased, and most of them changed into mature organized fibrocytes and arranged themselves along the repaired connective tissue. The number of blood vessels decreased, but their caliber was larger than in the case of untreated skin [39]. It is believed that the efficacy of *C. officinalis* in wound healing is primarily due to the content of flavonoids, which have an effect on reducing edema and regulating microcirculation [39].

### 3.13. Arnica Montana

*Arnica montana* is a plant from the *Asteraceae* family. It occurs in Siberia and mountainous European countries [223,224]. It is a perennial herbaceous plant. The unbranched aboveground parts reach about 15–60 cm in height. It has ovate leaves and small, light yellow flowers with a diameter of 6–8 cm [225]. They are most often used for medicinal purposes [223]. They are used to prepare ethanol extracts [226] as well as gels, ointments, and creams to apply to the skin in case of injuries and swelling [38]. *A. montana* contains flavonoids (e.g., quercetin) and phenolic acids (e.g., caffeic, ferulic, gallic, p-hydroxybenzoic acids) with antioxidant effects, as well as sesquiterpene lactones with anti-inflammatory effects [227]. In addition, Arnica flower is a rich source of, among others, essential oil, carotenoids, and phytosterols. Compounds contained in mountain Arnica penetrate deep layers of the skin, strengthen the walls of blood vessels, and prevent clots [227]. The mechanism of action of Arnica on blood vessels in the skin is not yet fully understood. Studies have shown that anti-inflammatory properties are caused by, among others, inhibition of histamine release from degranulated mast cells, reduction of prostaglandin production including thromboxane, and reduction of serotonin release from platelets. These actions are probably influenced by the high content of sesquiterpene lactones, including helenin and chamissonolide, which reduce swelling and petechiae through anti-inflammatory action [228]. This plant has antipetechial effects, increasing the activity of macrophages. Studies have shown a difference in the reduction of laser-induced bruising in healthy volunteers after the application of a preparation containing 20% Arnica compared with white petrolatum or a mixture of 0.3% retinol and 1% vitamin K [229]. *A. montana* is often used in the form of homeopathic medicines. However, a randomized trial conducted to evaluate the effect of oral administration of this plant on postoperative bruising after carpal tunnel surgery did not show a significant improvement in the appearance of bruises on the 4th day after surgery [230]. Similarly, no difference was found after local application of 10% Arnica ointment in 36 patients after blepharoplasty, after which one side was treated with Arnica ointment or placebo and the other side was untreated and served as a control. After 3 days, 7 days, and 6 weeks, there was no difference in the general appearance of the periorbital area regarding edema, petechiae, and erythema [167]. Meanwhile, a topical preparation containing *A. montana* and *Rhododendron tomentosum* (*Ledum palustre*) in 27 patients after undergoing typical ophthalmic procedures, including blepharoplasty, browpexy, and rhinoplasty, accelerated the healing of postoperative petechiae and edema [168].

### 3.14. Ruscus Aculeatus

R. aculeatus belongs to the Asparagaceae family. It is a small, 20–60 cm tall evergreen perennial subshrub [22,36,231]. It is highly branched with triangular leaves and greenish-white flowers of about 3 mm. It also has bright red, berry-shaped fruits with large seeds [231]. The striated stems grow to 1 m in length. It occurs naturally in the Mediterranean and southwestern European regions [232]. It contains, among others, steroid saponins (neuroruscogenin and ruscogenin), coumarins, flavonoids, sterols, glycolic acid, and triterpenes. Both aboveground and underground parts of the plant are used in medicine [22]. Alcoholic extracts reduce venous permeability and relieve swelling [36]. Additionally, local application of R. aculeatus shows anti-inflammatory effects [232]. The effects of this plant on microcirculation include its endothelial-protective effect, through antioxidant and anti-inflammatory effects, which consequently prevents leukocyte adhesion and their leakage into tissues. Flavonoids and ruscogenin are the main active substances thanks to which R. aculeatus has been used in venous system problems. Ruscogenin inhibits leukocyte migration by regulating both protein and mRNA. In a study, the venous constriction effect after local application of R. aculeatus extract was assessed. A randomized, double-blind study was conducted with 18 healthy volunteers. After application of 4 to 6 g of a cream containing 64 to 96 mg of an extract, the diameter of the femoral vein decreased by an average of 1.25 mm, while placebo was associated with a diameter increase of 0.5 mm (mean) (*p* = 0.014). The authors suggest that the reduction in the vein diameter reflects good percutaneous absorption of the active substances contained in butcher’s broom [169]. Ruscogenin also exhibits remarkable antielastase activity. R. aculeatus extract led to a significant reduction in the diameter of deep veins and, at the same time, to an increase in flow parameters but a statistically insignificant reduction in flow parameters in superficial veins in primary varicose vein disease [233].

The plants analyzed in this review are both used in traditional medicines and less popular ones, in which interest has increased in recent years. The greater interest in topical use of plants is also often due to the lower risk of adverse effects compared to oral use (Table 7).

It should be emphasized that in most cases in our review, the analyzed studies were preliminary studies. There are few reports in the available literature on the external use of plant preparations as blood vessel sealants. It should be noted that some of the analyzed studies were conducted in vivo—both on animals and with the participation of humans—which may suggest a potentially beneficial effect of these plant materials on the cutaneous vascular system. In most of the analyzed cases, plants/plant extracts or preparations prepared independently on the basis of plant materials were used. Therefore, a major limitation in this type of research is often the use of plant material, the composition of which may vary depending on many factors, e.g., seasonality, preparation methods including a given plant material, or extraction methods of the plant itself. Natural medicines are characterized by a certain limitation, mainly caused by the fact that plants are very rich sources of various secondary metabolites. Natural compounds interact with each other, which may make it difficult to predict the full therapeutic effect. On the other hand, synthetic drugs contain specific active substances, which usually show a faster and stronger therapeutic effect compared to substances derived from plants. Therefore, despite the many advantages of using natural raw materials in this area, the lack of a precisely repeated research protocol, especially clinical, is often a major limitation. In addition, the content of active substances in plants and the subsequent therapeutic effect are also limited by the sensibility of plants and growth in different climatic conditions, which have a huge impact on the content of active substances in them. On the other hand, the search for interesting new solutions using natural raw materials fits perfectly into the trend of naturalness, which has become very common in recent years.

## 4. Conclusions

This review attempts to characterize and discuss various plants rich in valuable secondary metabolites as well as to present their functions, applications, and mechanisms of action. Due to the small number of reports in the available literature on this subject, this work also describes the effect of plants on common ailments related to the condition of blood vessels in the skin. Undoubtedly, active compounds contained in plants have a great impact on the condition of blood vessels in the skin. Their effects include, among others, improving microcirculation, supporting the elasticity of vessels, or reducing inflammation. Plants rich in flavonoids, polyphenols, and other active substances contribute to improving blood flow, reducing swelling, and protecting against oxidative stress. As a result, regular use of plant preparations can support skin health, improve its appearance, and accelerate tissue regeneration, while maintaining the balance of vascular functions. Biocompounds are considered essential components of nutraceuticals, pharmaceutical products, and cosmetic ingredients due to their antioxidant and anti-inflammatory properties. These natural active substances can also constitute an important group of ingredients used in the prevention and treatment of circulatory system diseases. On the other hand, due to the small amount of research on this topic, further analysis is necessary to explain the deeper mechanism of action as well as to select those plants that will be safe and at the same time have a beneficial effect on the condition of blood vessels in the skin, which will help in preventive care and medical care.

## Figures and Tables

**Table 1:** Examples of traditional use of medicinal plants.

Plant	Family	Traditional Uses	Ref.
*Camellia sinensis*	*Theaceae*	Promoting the excretion of urine; controlling bleeding; helping heal wounds; improving heart health; regulating flatulence, body temperature, and blood sugar; promoting digestion; improving mental processes	[9]
*Chrysanthellum* *indicum*	*Asteraceae*	The treatment of common cold; fever; migraine; conjunctivitis; eye irritation; hypertension; inflammation; ulcerative colitis; vertigo; ophthalmia with swelling as well as skin infections	[10]
*Helichrysum* *italicum*	*Asteraceae*	Health purposes in the respiratory and digestive tracts as well as the skin; wound healing; antimicrobial uses; gall and bladder disorders; analgesic; common types of preparations are infusions, decoctions for both oral and external use, followed by vapors, juices, and powders	[11]
*Glycyrrhiza glabra*	*Fabaceae*	Respiratory disorders; hyperdipsia; epilepsy; fever; sexual debility; paralysis; stomach ulcers; rheumatism; skin diseases; hemorrhagic diseases; jaundice	[12]
*Artemisia annua*	*Asteraceae*	The treatment of jaundice; antibacterial; antipyretic agent in malaria; tuberculosis; wounds; hemorrhoids; viral, bacterial, and autoimmune diseases	[13]
*Aesculus* *hippocastanum*	*Sapindaceae Juss*	For flatulence; anorexia; antiseptic; antioxidant; antipyretic; analgesic and antiaging agent; in treatment of infections of ear, nose, and throat regions; source of non-edible starch and timber	[14]
*Potentilla erecta*	*Rosaceae*	Astringent agent to treat bleeding and inflammation of the skin as well as mucosa and diarrhea	[15]
*Achillea millefolium*	*Asteraceae*	An antihemorrhagic; wound-healing agent; remedy for ulcers, skin diseases (wounds); snakebites and varicose veins; skin inflammation	[16,17]
*Quassia amara*	*Simaroubaceae*	Antimalarial; stomachic; antianemic; antibiotic; cytotoxic; antiamoebic activity; reproductive function; insecticidal, larvicidal, and vermifuge properties	[18]
*Ginkgo biloba*	*Ginkgoaceae*	Relieve cough; reduce phlegm; clear poison and relieve diarrhea	[19]
*Artemisia* *lavandulaefolia*	*Asteraceae*	Various diseases; digestive; anthelmintic; effective odor remover; gastrointestinal diseases; constipation; pain; belly pain; asthma; gynecological problems	[2]
*Calendula officinalis*	*Asteraceae*	Gynecological issues; digestive disorders; inflammation of the oral and pharyngeal mucosa; eye conditions; skin injuries; burns; vision problems; menstrual irregularities; diaphoretic; blood purifier; helps lower blood sugar levels; tinctures, ointments, and balms; antitumor; astringent; diuretic; antipyretic; anti-inflammatory properties; wounds; acne; scars; herpes; frostbite; chickenpox; mumps; gangrene; ulcers; insect bites; boils; skin inflammation; toothaches; oral sores; gargles; varicose veins, soothing agents for diaper rash; hemorrhoids; conjunctivitis; mouth inflammation; homeopathy	[20]
*Arnica montana*	*Asteraceae*	Bruises; sprains; back pain; rheumatoid arthritis; phlebitis	[21]
*Ruscus aculeatus*	*Asparagaceae*	Diuretic effects; urinary tract disorders; laxative	[22]

**Table 2:** Examples of sources of flavonoids [78,79].

Flavonoid Class	Flavonoid Example	Source
Flavonol	Quercetin	Apples
Flavanol	Catechins	Teas
Isoflavone	Genistein	Soybeans
Flavone	Luteolin	*Ginko biloba*
Flavanone	Hestepridin	Oranges
Anthocyanin	Cyanidin	Blackberries

**Table 3:** Examples of sources of derivates of hydroxybenzoic acids and hydroxycinnamic acids [89].

Main Phenolic Acids	Derivative Phenolic Acid Examples	Source
Hydroxybenzoic acids	Gallic acid	*A. montana*
	Syringic acid	Açaí (Euterpe oleraceae Mart.)
	p-Hydroxybenzoic acid	Soybeans, *A. montana*
	Protocatechuic acid	*Ginkgo biloba*
Hydroxycinnamic acids	Ferulic acid	Corn
	Caffeic acid	Potato
	p-Coumaric acid	Tomato
	Sinapic acid	Celery

**Table 4:** Popular sources of carotenoids [55,151,156].

Carotenoid Class	Carotenoid Example	Source
Carotenes	β-carotene	Carrots
	Lycopene	Tomatoes
Xanthophylls	Lutein	Egg yolk
	Zeaxanthin	Watermelon
	Capsanthine	Peppers
	Astaxanthin	Crustaceans

**Table 5:** Effectiveness of different plant compounds.

Group of Active Compounds	Examples of Compounds	Effects of Different Plant Compounds	Ref.
Vitamins	Vitamin C	Collagen synthesis, strengthening vessels, improving circulation, antioxidant protection	[45,115]
	Vitamin E	Strong antioxidant, protection of blood vessels from damage, anticancer, photoprotective, and antiaging effect, skin regeneration	[123,124,125,126] [130,131,132,133,134,135] [136]
	Vitamin D	Reduction of atherogenesis	[137,138,139]
	Vitamin B2	Antioxidant, maintaining healthy blood vessel walls	[140,141,142,143]
	Vitamin B3	Improvement of endothelial function, lowering the level of bad cholesterol (LDL), increasing the level of good cholesterol (HDL)	[52,140]
	Vitamin B5	Improvement of endothelial function, lowering the level of bad cholesterol (LDL), increasing the level of good cholesterol (HDL)	[52,140]
	Vitamin B6	Lowering homocysteine levels	[140,141,143]
	Vitamin B7	Participation in maintaining balance	[140,141,143]
	Vitamin B9	Lowering homocysteine levels, influence on the regeneration of blood vessels	[140,141,143]
	Vitamin B12	Anti-inflammatory, antifibrotic, antiradiation effects, lowering homocysteine levels	[144,145]
Carotenoids	β-carotene	Participation in the photosynthesis process, protection against UV radiation and oxidative stress, influence on the hormonal balance of plants	[32,153]
	Lutein		
	Zeaxanthin		
	Lycopene		
Polyphenols	Flavonoids	Antioxidant, anticancer, anti-inflammatory, and antimutagenic effects improving the elasticity of blood vessels	[64,65,66,67,68] [44,70,71,72]
	Phenolic acids	Anti-inflammatory, antiallergic, antimicrobial, antioxidant, antithrombotic, cardioprotective, anticancer, hepatoprotective, and antidiabetic effects	[80,81,82,83,84,85,86]
	Stilbenes	Anti-inflammatory, anticancer, and antioxidant effects, prevention of excessive platelet aggregation, UVB skin protection, collagen synthesis, inhibition of melanogenesis	[49,90,91,92,93,94,95,96,97,98]
	Lignans	Antioxidant, anti-inflammatory, and antiaging effects, estrogen-modulating effect	[28,72,103,104,105,106,107,108]
Saponins		Diuretic, emetic, expectorant, and antiseptic effects	[15,25,27,54,149]

**Table 6:** Selected plants affecting blood vessels in in vitro and in vivo studies.

Plant	Study Model	Dose/Treatment Time	Results/Effect	Ref.
***Camellia sinensis***	In vivo randomized, double-blind, split-face trial four volunteers, age 40–59 years with significant erythema and telangiectasia In vivo double blind, placebo-controlled trial, 40 women with moderate photoaging	A cream containing 2.5% *w*/*w* of epigallocatechin gallate applied on the skin face/6 weeks Topical 10% green tea and oral 300 mg green tea supplementation for clinical and histological purposes/twice daily as well as placebo for 8 weeks	Angiogenic growth, vascular endothelial growth factor (VEGF), and hypoxia-inducible factor-1 (HIF-1) Reduction in visible telangiectasia (no significant differences)	[157] [158]
***Chrysanthellum indicum***	In vivo multicenter randomized, double-blind, parallel group, placebo-controlled study, 246 people aged 18–80 with clinically moderate rosacea	A cream containing 1% extract of *Ch. indicum* and placebo/12 weeks, applied twice a day	Reduction in erythema score (41.3%), overall rosacea severity compared to baseline and placebo	[159]
***Helichrysum italicum***	In vivo double-blind, placebo-controlled in vivo study, 43 women aged 30–65	*H. italicum* extract on the legs (5%), the eyes (3%), and the face (1%)/applied for 28 days twice daily	Reduction of red spots and visible capillaries	[41]
***Glycyrrhiza glabra***	In vivo study, 62 women with mild to moderate red facial skin, mean age of 48 years	The products contained extract from the licorice root of *Glycyrrhiza inflata*/8 weeks	Significant improvements in average erythema scores were observed at 4 and 8 weeks	[160]
***Artemisia annua***	In vivo study, mouse models with rosacea-like inflammation, 25 male mice aged 7 weeks In vivo randomized, investigator-blinded, parallel-group study, 130 patients with papulopustular rosacea	Treatment groups gavaged with 25, 50, or 100 mg/kg artesunate solution/7 weeks Emulsion with artemisinin derivative (1%) or metronidazole emulsion (3%)/4 weeks with observation up to 8 weeks	Reduction of erythema, number of inflammatory cells, and myeloperoxidase activity (MPO) Reduction of erythema, papules, and pustules and good tolerance of the cream with artemisinin derivative by patients	[161] [162]
***Aeskulus hippocastanum***	In vitro study, flow cytometric studies of adhesion and molecule expression, determination of proinflammatory cytokines IL-6 and IL-8 in the extracellular environment	Isolated compounds from *A. hypocastanum*	Inhibition of IL-6 and IL-8 secretion and blockage of the VEGF proangiogenic factor	[163]
***Potentilla erecta***	In vitro study, human keratinocyte cell line HaCaT in occlusive patch test and a collagen contraction assay In vivo randomized, prospective, placebo-controlled, double-blind study, 40 healthy non-smoking individuals of both genders, age 18–54 years, skin phototypes II and III	*P. erecta* extract enriched argrimoniin *P. erecta* cream (2%) compared to 1% hydrocortisone acetate/twice daily for two weeks	Reduced IL-6 and PGE2 or NF-κB activation in cells, narrowing of blood vessels Reduction of erythema with atopic skin. In the UV erythema test, PO cream significantly reduced the erythema index compared to the vehicle. The anti-inflammatory effects of PO cream were comparable to those of 1% hydrocortisone acetate cream	[15] [26]
***Achillea millefolium***	In vivo study, liver incision and histopathological analysis, 12 female Wistar rats	*A. millefolium* methanolic extract (150 mg/kg)/analysis after 4, 6, and 8 weeks	Significantly reduced bleeding time, histopathological analysis showed no signs of toxicity or hepatic damage after 4, 6, and 8 weeks in the female rats	[16]
***Quassia amara***	In vivo study, local application of the preparation, 30 persons with rosea	*Q. amara* gel (4%)/twice a day for 6 weeks	Reduced flushing, erythema, telangiectasia	[164]
***Ginkgo biloba***	In vitro study with human umbilical vein endothelial cells (HUVECs) of the signaling triggered by VEGF	Kaempferol identified in *G. biloba*	Positive effects of kaempferol on macrophage migration and tissue regeneration via modulation of macrophages via the VEGFR signaling pathway	[165]
***Artemisia lavandulaefolia***	In vitro study, human epidermal keratinocytes In vitro study, human umbilical vein endothelial cells (HUVECs)	The effect of chlorogenic acid isomers isolated from *A. lavandulaefolia* on the expression of KLK5 in HEKn and extracellular enzymatic activity The effect of *A. lavandulaefolia* extract on the formation of the HUVEC blood vessel network	Inhibition of KLK5 protease activity Inhibited HUVEC tubule formation, inhibited HUVEC migration and invasion, inhibited angiogenesis	[166] [2]
***Calendula officinalis***	In vivo study histopathological and biochemical tests, 75 white male rats	Gel containing *C. officinalis* extract (5%, 7%, and 10%)/14 and 21 days	On day 14, the wound size decreased in all groups and on day 21 after injury the wound size in the treated lesions was smaller than in the control	[39]
***Arnica montana***	Randomized, placebo-controlled trial, 36 patients In vivo after undergoing common oculofacial procedures, including blepharoplasty, browpexy, and rhinoplasty, 27 patients	Arnica ointment 10%/6 weeks Local hydrogel pads containing *A. montana* 50%/up to 6 days after surgery	There were no significant differences in the overall periorbital appearance regarding edema, petechiae, or erythema Reduction of postoperative petechiae and edema after ophthalmic and facial procedures	[167] [168]
***Ruscus aculeatus***	In vivo randomized, double-blind study, 18 healthy volunteers	Application of a preparation containing 64 to 96 mg of *R. aculeatus* extract	Reduction in the diameter of the femoral vein	[169]

**Table 7:** Summary of the local and oral effects of selected plants with a positive effect on blood vessels in the skin.

Criterion	Topical Use	Oral Use	Ref.
**Action**	Topical effect, application of the preparation on the skin in the disease area	Systemic effect	[158,159,162,164,193,234,235,236]
**Effectiveness**	Positive effect on skin lesion, e.g., rosacea, erythema	Wide application in various diseases and disorders of body functions—systemic effect	
**Dosage**	Low risk of overdose because the absorption of active ingredients is reduced	Risk of exceeding the recommended dose and occurrence of side effects	
**Side effects**	Irritation	Increased risk of side effects by exceeding the recommended dose (e.g., hepatotoxicity)	
**Effects on the digestive system**	No effect on the digestive system	Gastrointestinal disorders	
**Examples of herbs used**	*Chrysanthellum indicum, Quassia amara, Aeskulus hippocastanum*	*Camellia sinensis, Artemisia annua, Aeskulus hippocastanum*	

## Data Availability

Not applicable.
